# Metabolite profiling and protein quantification to a large library of 96 horsegram (*Macrotyloma uniflorum*) germplasm

**DOI:** 10.1038/s41598-022-11962-7

**Published:** 2022-05-12

**Authors:** Manisha Gautam, Rakesh Kumar Chahota

**Affiliations:** Department of Agricultural Biotechnology, CSK Himachal Pradesh Agricultural University, Palampur, H.P 176062 India

**Keywords:** Environmental chemistry, Natural variation in plants, Plant breeding, Biogeochemistry, Biomarkers

## Abstract

The present study was framed for the assessment of metabolic diversity of 96 diverse horsegram lines derived from 700 germplasm accessions of Pan India. The nutritional component of horsegram germplasm was studied using nuclear magnetic resonance spectroscopy (^1^H NMR) and micro-Kjeldal method. Differential range of protein content was found 13–40% in the selected panel. The related wild species *Macrotyloma sar-gharwalensis* contained highest protein content (40%), and the lowest protein content (13%) was found in IC-120837 and TCR-1439. Seed based ^1^H NMR spectroscopy of horsegram discovered 45 different metabolites (17 amino acids, 7 flavonoids, 10 organic acids/phenolic acids/sugar alcohols/steroids, 7 carbohydrades/sugars, and 4 vitamins). The number of total metabolites quantified among diverse horsegram panel ranged from 25–44. The lowest metabolites number was 26 in PLKU-38, while, IC-280031 and IC-139356 lines showed the presence of highest number of metabolites (44). Lines IC-280031 and IC-139356 were found most nutritive amongst selected panel of horsegram germplasm by containing maximum number of quantifiable metabolites during ^1^H NMR spectroscopy study. Further, the NMR based data of 96 germplasms was subjected for statistical analysis (PCA, Matrix plot, stacked charts) that revealed the similarities and variations among the whole germplasm. The methionine, sucrose, maltose, riboflavin and myricetin were observed as differential chemo-markers that help to differentiate the horsegram lines of selected panel. This information will further aid in the selection of nutritionally efficient cultivars for proteomics and genomics studies and these lines can be used as nutraceutical food for the prevention of various health ailments including kidney stone.

## Introduction

The role of germplasm collections and its evaluation for agronomic and nutritional traits is the integral part of cultivars improvement programme in developing countries as many landraces still retains their specific traits in stable way though propagation is still limited despite of its worldwide recognition^1–3^. Germplasm collection covers the whole process of evaluation of genotypes, preliminary studies if any, characterization of plants and the documentation starts from obtaining the new samples, growing them for data generation and multiplication. The working collections of germplasm include only the quality genotypes having good yield and other major traits like disease resistance, pest’s resistance and stress tolerance therefore many other desirable alleles for less important traits may be ignored^4^ highlighted the importance of germplasm evaluation in some major crops of India and described the guidelines for evaluation of diverse lines.

In the recent years, the nutraceutical potential of cultivated plants and their visible benefits in food science has been developing day by day. Furthermore, the analysis of entire composition of the food expresses the connecting varietal characteristics difference by focusing on particular constituents in it^5^. Metabolic changes express the plant developmental processes and their adaptation in response to abiotic stresses, while the metabolic composition is depending upon the quality traits of plants. Also the metabolic information gives very accurate biological information more than other proteomics studies. This information has been achieved by application of advanced analytical techniques and various other omics tools^6^. Among the available omics tools, nuclear magnetic resonance (NMR) spectroscopy is unbiased and rapid technology to analyse large sample size. NMR spectroscopy can also be used to analyse several components in a food mixture at the same time with limited sample destruction^7^. This technique is highly reproducible and helps in quantitative studies, which makes this technique more advantageous than others. In addition, this approach can be used to study the varietal difference of metabolites and also provides strong evidence for determining the quality, originality and taste evaluation during selection of suitable cultivar^8^. NMR metabolic profiling is useful for systematic analysis of metabolome/metabolites and quality control studies for agricultural and biotechnological products^9^.

Horsegram (*Macrotyloma uniflorum* (Lam.) Verdc.) is a lesser-known leguminous crop despite of its good drought and saline tolerant capabilities its cultivtion is not spreading to new areas. In addition it also possess numerous nutraceutical traits such as antioxidant activity, radical scavenging behavior, and antihypertensive activity. Besides it is a rich source of protein, fiber and flavonoids. The nutraceutical value of horsegram in the diet is unbeatable, especially for vegetarians in developing countries like India, where most of the population suffers from protein malnutrition. However, the insufficiency of metabolomics studies hampered the selection process of high yielding, nutritionally rich and stress tolerant germplasm in conventional breeding programmes. Thus, horsegram genotypes considered for this study procured form various agro ecological zones of India and are classified based on metabolic characterstics. A micro-Kjeldal procedure and nuclear magnetic resonance (NMR)-based metabolomics approach were used to investigate the variations in protein and metabolites among the diverse horsegram germplasm lines^9^. Furthermore, no study has been reported at national or at global level on metabolic characterization of different accessions of horsegram germplasm using NMR, on which these lines can be exploited for nutraceutical uses. In the present study we used NMR spectroscopy in conjunction with protein analysis through the micro-Kjeldal method to provide good and comprehensive information about the compositional differences among commercially important indian horsegram lines. The aim of this research is to identify genotypes with higher metabolite concentration, number of quantifiable metabolites and total protein content to develop biofortified varieties, which can be used as nutraceutical food.

## Materials and methods

Diverse genotypes procured from different sources such as NBPGR (National Bureau of Plant Genetic Resources), New Delhi and various regional research stations of NBPGR. Personal collection from North western Himalayan regions were made by following the institutional guidelines and national legistation and these lines are being maintained in the Department of Agricultural Biotechnology, CSK Himachal Pradesh Agricultural University, Palampur, Himachal Pradesh, India. After removing the duplicates 360 lines selected from 520 lines were evaluated for various morphological traits for two consecutive year and molecular markers also employed to assess the diversity at molecular level. Using diversity analysis and PowerCore software, 96 diverse lines (Table SI-1) were selected for further metabolomics and total protein analysis. The evaluation experiments involving plants were performed in accordance with relevant guidelines and regulations. The variation for selected panel was assessed on the basis of 12 morpho-agronomical characters and the genotype with desirable genetic variation for number of pods per plant, days to maturity and plant height, number of seeds per plant, 100 seed weight, seed yield per plant were included in the core panel^10^.

### Chemicals

All the chemicals including internal standard TSP (3-(Trimethylsilyl)-1-propanesulfonic acid) used in the study were of analytical grade (Sigma-Aldrich and Merck, Mumbai, India).

### Protein determination

The estimation of total protein (crude) was done by using micro-Kjeldahl method. The amount of protein present in the seeds was calculated from the nitrogen concentration present in it. For the experiment, seeds of each line were taken and the total protein content was calculated using nitrogen conversion factors of 6.25. The seed sample (200-250 mg) in 3gm of digestion mixture (K_2_SO_4_:CuSO_4_ in 10:1 ratio) with 10 ml of sulfuric acid was digested at temperature higher than 400 °C for 1–2 h. Organically bonded nitrogen in the whole process of catalytic conversion was converted into ammonium sulfate. Further, ammonia was liberated after the alkalization of the digested solution, whose steam distillation was done and concentration was determined by titration against 0.1NHCl^11^.

## Metabolite profiling using NMR spectroscopy

### Sample preparation

The seeds of each line of selected panel were ground to fine powder and sonicated for two times with 80% methanol for 30 min at 45 °C. All extracts of the individual samples were combined and dried under reduced pressure in a rotary evaporator. The respective extracts were used for the NMR study. All test samples (500 mg) and internal standard TSP (0.7 mg) were dissolved in 0.7 mL NMR-grade solvent, CD_3_OD and D_2_O (80:20), and then transferred into a 5-mm NMR tube for NMR analysis^12–14^.

### Data processing

Fourier Transform (FT)-NMR data processing software (Bruker’s TopSpin™ software version 4.0) was used for ^1^H NMR spectral analysis. Microsoft Excel and Past 4.0 software were used for the calculation of correlations among the different lines with the use of identified metabolites.

### NMR spectral analysis

NMR frequency of 600 MHz with a 600-MHz Bruker AVANCE III instrument was used for ^1^H NMR spectral analysis at 25 °C. Moreover, the identity of metabolites was confirmed by correlation spectroscopy (COSY). Further, the concentrations of the identified metabolites were calculated using known internal standard, TSP^12–14^ using following formula:

$$m_{{\text{X}}} = m_{{{\text{ST}}}} \times (A_{{\text{X}}} /A_{{{\text{ST}})}} \times \left( {{\text{MW}}_{{\text{X}}} /{\text{MW}}_{{{\text{ST}}}} } \right) \times \left( {N_{{{\text{ST}}}} /N_{{\text{X}}} } \right);$$

where *m*_X_ is the target analyte mass (unknown), *m*_ST_ is the internal standard mass, *A*_X_ and *A*_ST_ are the selected signals integral areas, *N*_X_ and *N*_ST_ are the integral signals generated for the number of proton and MW_X_ and MW_ST_ are the molecular weights of targeted analyte and internal standard].

## Results and discussion

### ^1^H NMR spectroscopy of seed metabolites

The seeds of all selected 96 lines of horsegram germplasm were analyzed using ^1^H NMR spectroscopy. The signals observed in 1D and 2D NMR spectral of the samples were compared with earlier established chemical signature for the *M. uniflorum* seeds^14^. This data profile was also confirmed through online databases such as HMDB (Human Metabolome Data Base), the Madison Metabolomics Consortium Database (MMCD), and the Biological Magnetic Resonance Bank (BMRB). The spectral data obtained from ^1^H NMR experiment was normalized, calibrated and bucketed into small spectral buckets (0.04 ppm). The ^1^H NMR spectral informations of *M. uniflorum* seeds were divided into three distinct regions [between *δ* 0.0–9.0 ppm: *δ* 0.0–3.0 ppm (fatty acid, amino acids, lipids, steroids), *δ* 3.0–6.0 ppm (sugars, polyols), and *δ* 6.0–9.0 ppm (Ketones, aldehydes, vitamins, polyphenols including flavonoids)].

### Distinct assignments and proton signals of ^1^H NMR spectral for different metabolites

**Figure 1:**
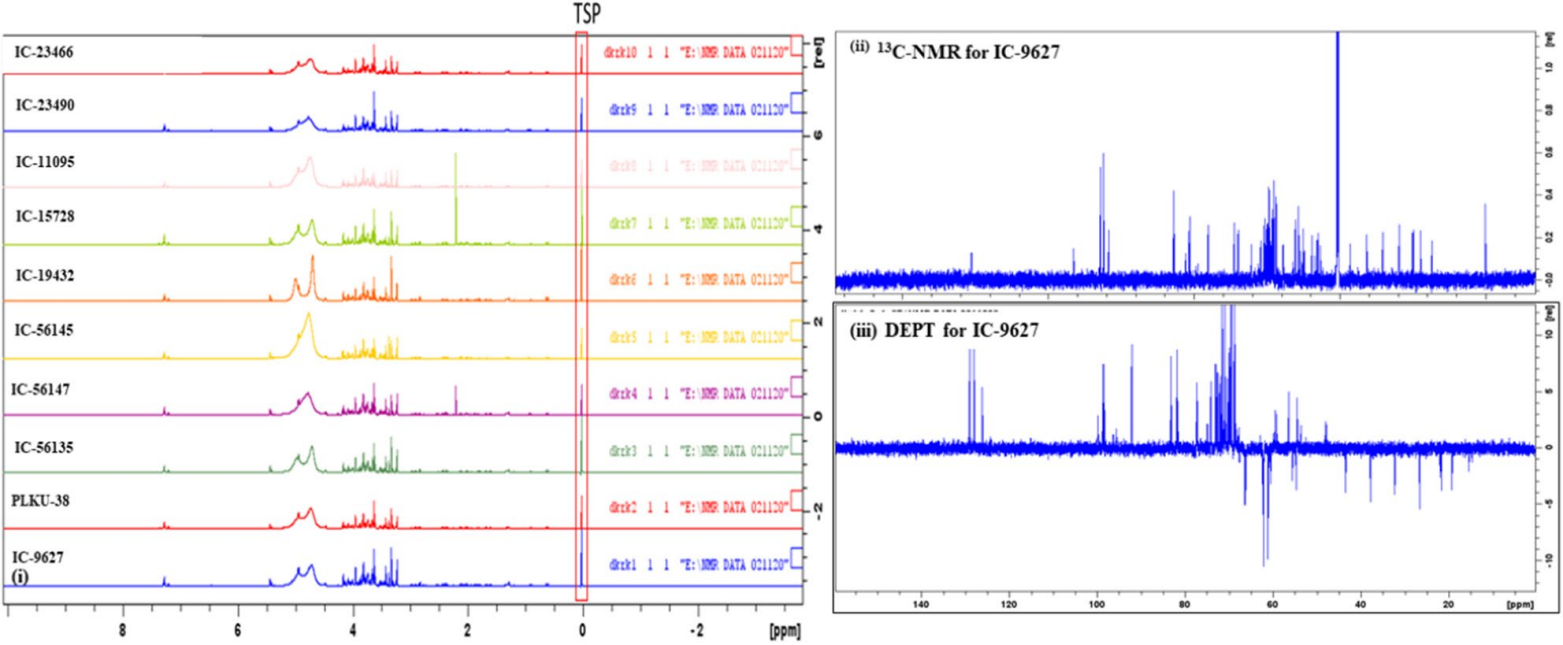
(**i**) Nuclear magnetic resonance spectral of ten representative lines of diverse panel of horsegram germplasm (**ii**)^13^C NMR spectra and (**iii**) Distortions Enhancement by Polarization Transfer (DEPT) spectrum for distinguishing between CH_3_ group, CH_2_ group and CH groups.

The NMR characteristic peak signals were assigned to selective metabolites of diverse panel of 96 lines to establish chemical signatures and spectral information of reference molecules (Fig. 1, Figure SI-1). The NMR spectral information showed spectral similarities and differences on comparison. The initial spectral information revealed intense signals for amino acids, phenolic acids, flavonoids and vitamins while overlapped signals for sugars. Some signals also had low intensity that was difficult to assign for metabolites. Therefore, 2D-NMR experiments *i.e.* HSQC (Heteronuclear Single Quantum Coherence), COSY (Correlated Spectroscopy), HMBC (Heteronuclear Multiple Bond Coherence), and NOESY (Nuclear Overhauser Effect Spectroscopy) were used with 1D-NMR (^1^H & ^13^C NMR) and Distortions Enhancement by Polarization Transfer (DEPT) spectrum for distinguishing between CH_3_, CH_2_ and CH groups to confirm the metabolites assignments (Fig. 1 and Figure SI-2). This resulted in the identification of a large set of metabolites (carbohydrates/sugars, organic acids, vitamins, phenolic acids, steroids and polyphenols/flavonoids) in the horsegram germplasm seeds. In total, 45 different metabolites from the chemical signature were chemically profiled, identified and quantified in the respective seed extracts of all selected 96 lines of horsegram germplasm (Table SI-2).

### Amino acids and sugars

**Figure 2:**
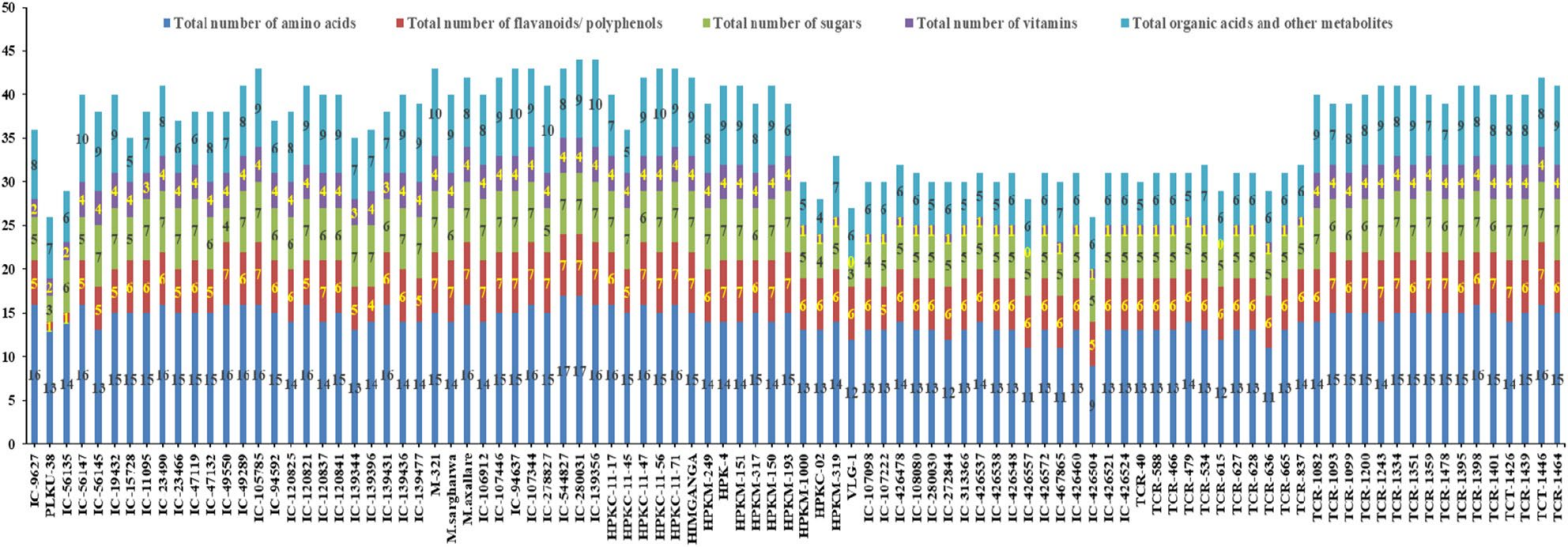
Distribution of different number of identified metabolites (amino acids, flavanoids/polyphenols, sugars, vitamins and organic acids and other metabolites) in the diverse panel of horsegram germplasm.

The amino acids were identified based on ^1^H NMR signals. The distinct assignments were assigned and confirmed from COSY experiment. The resonance of the peaks for amino acids were isoleucine (Ile) *δ* 0.88 (d), leucine (Lu) *δ* 0.97 (d), valine *δ* 1.06 (d), threonine (Thr) *δ* 1.320 (d), alanine *δ* 1.466(d), glutamine (Glu) *δ* 2.04(m), methionine (Me) *δ* 2.14 (CH_3_), aspargine (Asp) *δ* 2.85 (m), lysine (Lys) *δ* 3.03 (t), glycine *δ* 3.57 (s), glutamic acid (GluA) *δ* 3.74 (dd) serine (Sr) *δ* 3.95(m; 3.9440–3.9520), cystine *δ* 4.13 (dd), phenylalanine (Ph) *δ* 7.4176 (m; 7.4104–7.41076), tryptophan (Try) *δ* 7.72(d) and histidine *δ* 7.89 respectively (Table SI-2). Among the selected horsegram genotypes analyzed, the IC-544827 and IC-280031 lines have the highest abundance of amino acids (total 17) and IC-426504 line contained only 9 amino acids (Fig. 2) among all the quantified amino acids. The characteristic peaks of sugars and polyols were lies in range of *δ* 3.0–6.0-ppm in the ^1^H NMR spectral lines. The signals assigned to anomeric protons were at *δ* 4.06 ppm (d) (fructose), *δ* 4.57 ppm (d) (*β*-glucose), *δ* 5.13 ppm (d) (*α*-glucose)*, δ* 5.22 ppm (d) (_D_-xylose), *δ* 5.25 ppm (d) (*α*-galactose), and *δ* 5.42 ppm (d) (sucrose/Stachyose), respectively. The other grouped peaks represented the protons of other molecules in the range of *δ* 3.30–4.10 ppm [(inositiol at *δ* 3.49 (dd) and maltose at *δ* 3.88 ppm (dd) (Table SI-2)].

### Polyphenols and miscellaneous compounds

Polyphenols including flavonoids are important metabolites that plays a significant role in the functioning of plant growth in biotic and abiotic stresses. They have diversified health benefits. The polyphenols (phenolic acids and flavonoids) showed their presence between the range of *δ* 6.0–9.0. The phenolic acids/organic acids were assigned to the NMR signals at *δ* 5.35(m) [chlorogenic acid), *δ* 6.88(d) [caffeic acid], *δ* 6.91(d) [protocatechuic acid], and *δ* 6.99 (s) [gallic acid]. The signals at *δ* 6.20 (d) and 6.21(d) were representative of quercetin conjugates, while *δ* 6.51(d), *δ* 6.579(s), *δ* 6.656(s), and *δ* 6.8561 showed the presence of apigenin derivatives or its glycosides. Whereas, rutin, kaempferol glycoside, myricetin, quercetin and quercetin-3-O-glactoside were resonate at the characteristic chemical shift of *δ* 6.39(d), *δ* 7.15(d), *δ* 7.25 (s), *δ* 7.59(d) and *δ* 7.69 (d), respectively. Further, organic acids such as malic acid, succinic acid, quinic acid were observed at *δ* 2.37(dd), *δ* 2.542(s) and *δ* 1.83/1.96(d). The vitamins are also an essential nutrient and present in most of the pulses. The *M. uniflorum* also showed the presence of vitamins such as vit. B1 (Thiamine), vit. B2 (Riboflavin), vit. B3 (Niacin) and vit. E (Tocopherol) at their characteristic chemical shifts of *δ* 8.04 (s), *δ* 7.31 (s) and *δ* 8.80 (dd), respectively (Table SI-2). The germplasm lines namely; IC-139356, IC-278827, IC-94637, M-321, HPKC-11–56 and IC-56147 were found enriched in total polyphenols. The miscellaneous compounds that help in the plant survival under tough environmental conditions such as plant sterols, organic acids and choline were also identified. The chemical shifts at *δ* 1.04 (s) represented the steroids i.e. stigmasterol, *δ* 3.23/*δ* 3.27 (s) for choline. The characteristic peak assigned for fatty acid is at *δ* 0.625(Table SI-2).

### Qualitative comparison of metabolites screening in selected panel

**Figure 3:**
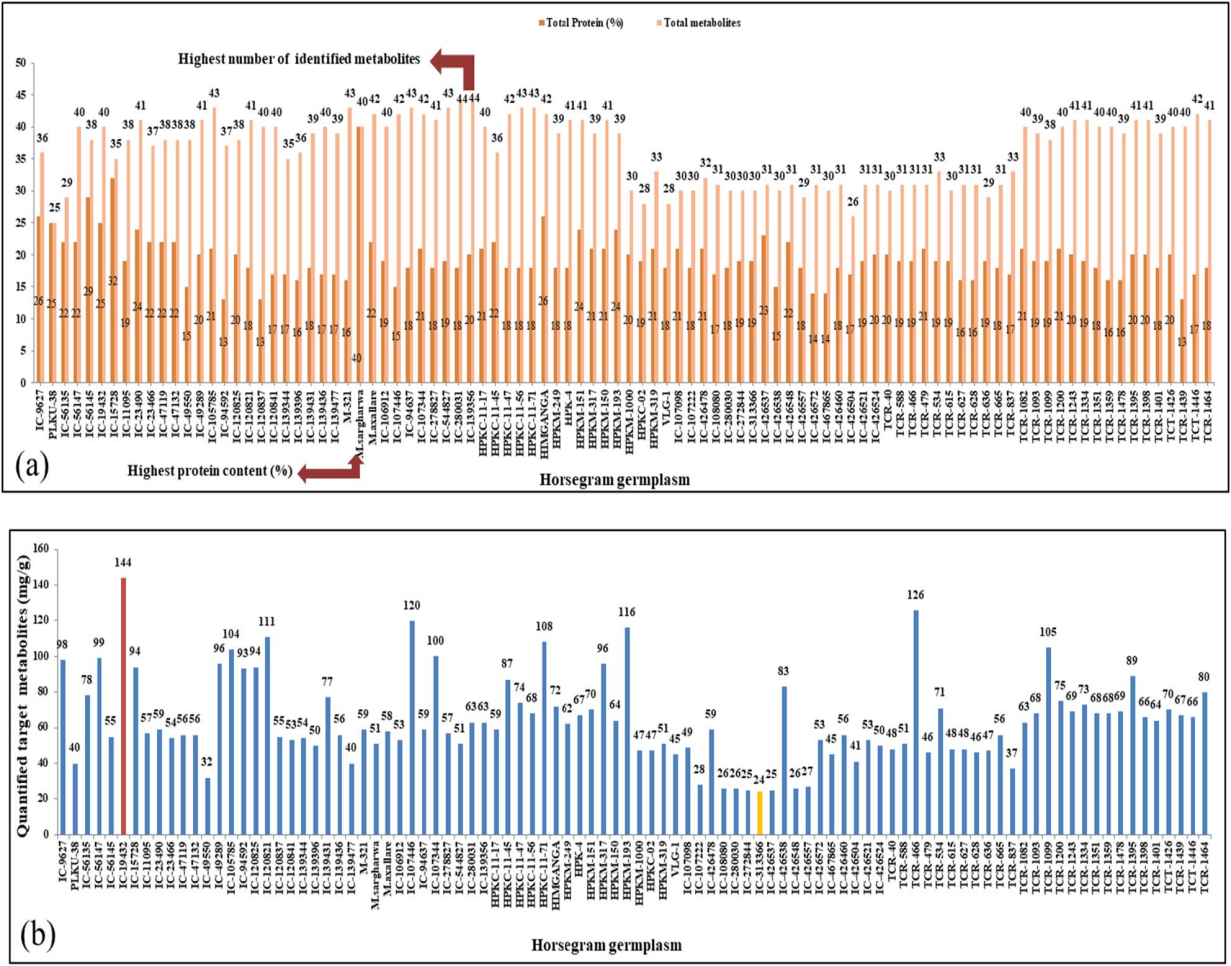
**(a)** Distribution of total protein and targeted metabolites among the horsegram germplasm [in terms of total number of metabolites identified and total % protein] (b) Quantification of identified metabolites in whole horsegram germplasm.

Qualitative screening of horsegram germplasms by ^1^H NMR spectroscopy has identified 45 chemically diverse metabolites in different germplasm samples of *M. uniflorum* seeds. The diversified metabolites consisting of amino acids, polyphenols (flavanoids and phenolic acid), organic acids, steroids, vitamins, fatty acids, choline. The numbers of total metabolites were ranged from 25–45 among the horsegram germplasm under study. Thirty lines contained comparable numbers of total metabolites *i.e.* ranged from 40–43. The lowest number of metabolites was found in the line namely PLKU-38 (26) (Fig. 3a) while, lines IC-280031 and IC-139356 were found nutritionally strong as these contained maximum number (44) of targeted metabolites quantified by ^1^H NMR.

Lines, IC-139356, IC-278827, IC-94637, M-321, HPKC-11–56 and IC-56147 were found rich in total organic acids, phenolic acids, sugar alcohols and steroids in whole core set. Some metabolites like amino acid (methionine), sugars (sucrose and maltose), vitamin (riboflavin) and flavonoid (myricetin) were determined to be biomarkers responsible for metabolic discriminations among the selected panel of horsegram germplasm. Total of 17 amino acids quantified in seeds of all horsegram accessions. The amino acid methionine was present in all the lines of the horsegram germplasm except IC-426504. Of the total identified sugars (07), two sugars sucrose and maltose were present in all lines of horsegram germplasm. The lines VLG-1, TCR-615 and IC-426557 contained no vitamins in their seed extracts in quantifiable amount (Fig. 2). Drought, heat, heavy metals and salinity like conditions are main stress factors that influence plant physiology with stimulation effect on secondary metabolite production in most of the crops and medicinal plants^15^. The variations observed in our study may be due to the factors cited by Isah^15^. Similar variations were also observed for metabolites evaluated by Radusiene et al.^16^ in *Hypericum perforatum* under different physiological conditions like temperature and light intensities. Further, the core line HPKC-02 contained only stigmasterol, succinic acid, choline and inositol. Amongst the identified flavonoids, myricetin (M) was found in all selected lines except IC-19432.

### Quantitative comparison of metabolites screening in selected panel of horsegram germplasm

The higher amount of total amino acids (45.2 mg/g) was reported in line TCR-466 and the maximum quantity of flavonoids (43.51 mg/g) were present in IC-56147 line. The line IC-19432 was found best for the sugar, sugar alcohols, vitamins, organic acids steroids and phenolic acid contents as it consists of highest amount (144 mg/g) of total quantified metabolites (Fig. 3b, Table SI-2) and contained adequate amount of all quantified amino acids (32.28 mg/g) accept two (lysine and histidine) that were not present in measurable quantity in this line.

IC-19432 line of horsegram germplasm contained all seven sugars (fructose, β-glucose, α-glucose*,*_D_-xylose, α-galactose, sucrose/Stachyose, inositol and maltose), four vitamins [riboflavin (Vitamin B2), thiamine (Vitamin B1), niacin (Vitamin B3) and tocopherol (Vitamin E)] and total ten organic acids steroids and phenolic acid including sugar alcohol [stigmasterol (St), quinic acid (QA), malic acid, succinic acid, choline (Ch), inostiol (In), chalorogenic acid, caffeic acid (CA) and gallic acid (GA)] in higher amount (24.95 mg/g, 14.47 mg/g and 30.97/g respectively). The protocatechuic acid (ProA) was found absent in this line (Fig. 3). Therefore, the three lines can be used as the biofortified variety for different purpose or can be incorporated in the breeding programme to develop tailored made biofortifed cultivars. Most of the time horsegram cultivated on marginal and drought prone area and lines identified for higher flavonoids and cinnamic acid may have higher drought tolerant capability as suggested by many researchers. The combination of flavonoids and cinnamic acid derivatives increased drought resistance in cotton plants, implying that they are highly effective at radical scavenging^17^. Isoprene generation in reed plants during heat stress suggests its efficient oxygen quenching antioxidant potential^18–20^. Flavonoids, terpenoids, and other volatile secondary metabolites give plants their colour and smell, which has repellent and attractive effects on insects and herbivores, while toxins can play a role in plant-plant allelopathic interactions^21,22^. In *Hypericum brasiliense* and garden pea, the abundance and volume of phenolic compounds biosynthesized were dramatically increased when the plants were grown under drought stress relative to the control^23,24^. Temperature fluctuations may have several effects on the expression of metabolic processes involved in the development of secondary metabolites in plant cells, tissues, and organs by affecting the morphology and metabolism of the plants through regulation, permeability, and intracellular reaction rate^15^.

### Comparison of protein screening in selected panel of horsegram germplasm

Total protein content in seeds of entire horsegram germplasm core set (96 lines) was estimated which revealed the presence of crude protein (13–40%) in the horsegram germplasm. The lowest value of protein was 13%, corresponding to IC-120837 and TCR-1439 (Fig. 3a). Though, the highest protein content was 40% among the entire germplasm of 96 lines, which was present in only one line namely *M. sar-gharwalensis*. Comparatively good amount of protein was found in other six lines, viz. IC-15728 (32%,), IC-56145 (29%), IC-9627 (26%) and almost similar protein content (24%) was present in three lines; IC-23490, HPKM-151 and HPKM-193. The related protein content may indicate that this underutilized legume has comparable nutritional value in terms of protein digestibility; however, more research is further needed.

Kawsar et al.^25^ used UV, IR, ^1^H NMR, ^13^C NMR and mass spectroscopy techniques to separate the aerial sections of *Macrotyloma uniflorum* Linn. Furthermore, when mouse erythrocytes were used to test its fractionated crude extract from 1-butanol fraction, it showed strong hemolytic activity but the seed part of *Macrotyloma uniflorum* Linn was not included in their study. Similar findings were observed in another study by Gautam et al.^14^ while working on *M. uniflorum* seed fractions using ^1^H NMR but the study included horsegram varieties from one state only (Himachal Pradesh, India).

### Multivariate statistical analysis for best chemotypes identification

**Figure 4:**
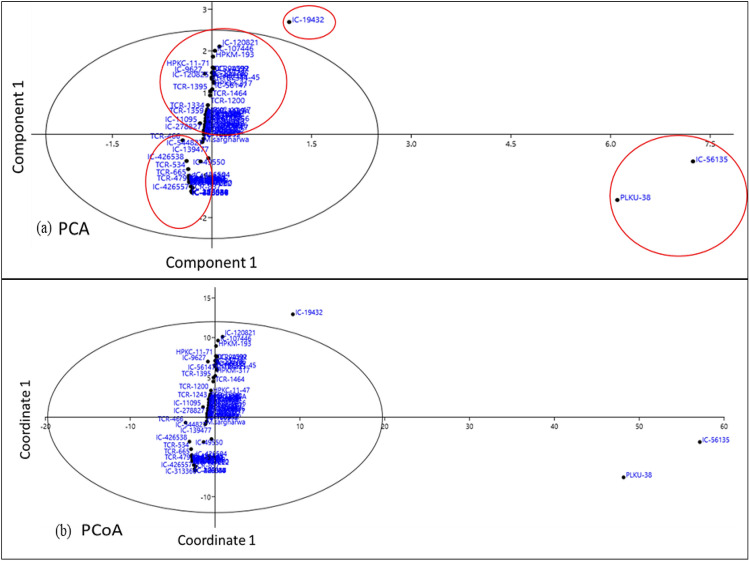
(**a**) PCA and (**b**) PCoA for diverse panel of horsegram germplasm on the basis of the identified metabolites. *PCA: Principal components analysis, PCoA: Principal coordinates analysis.

**Figure 5:**
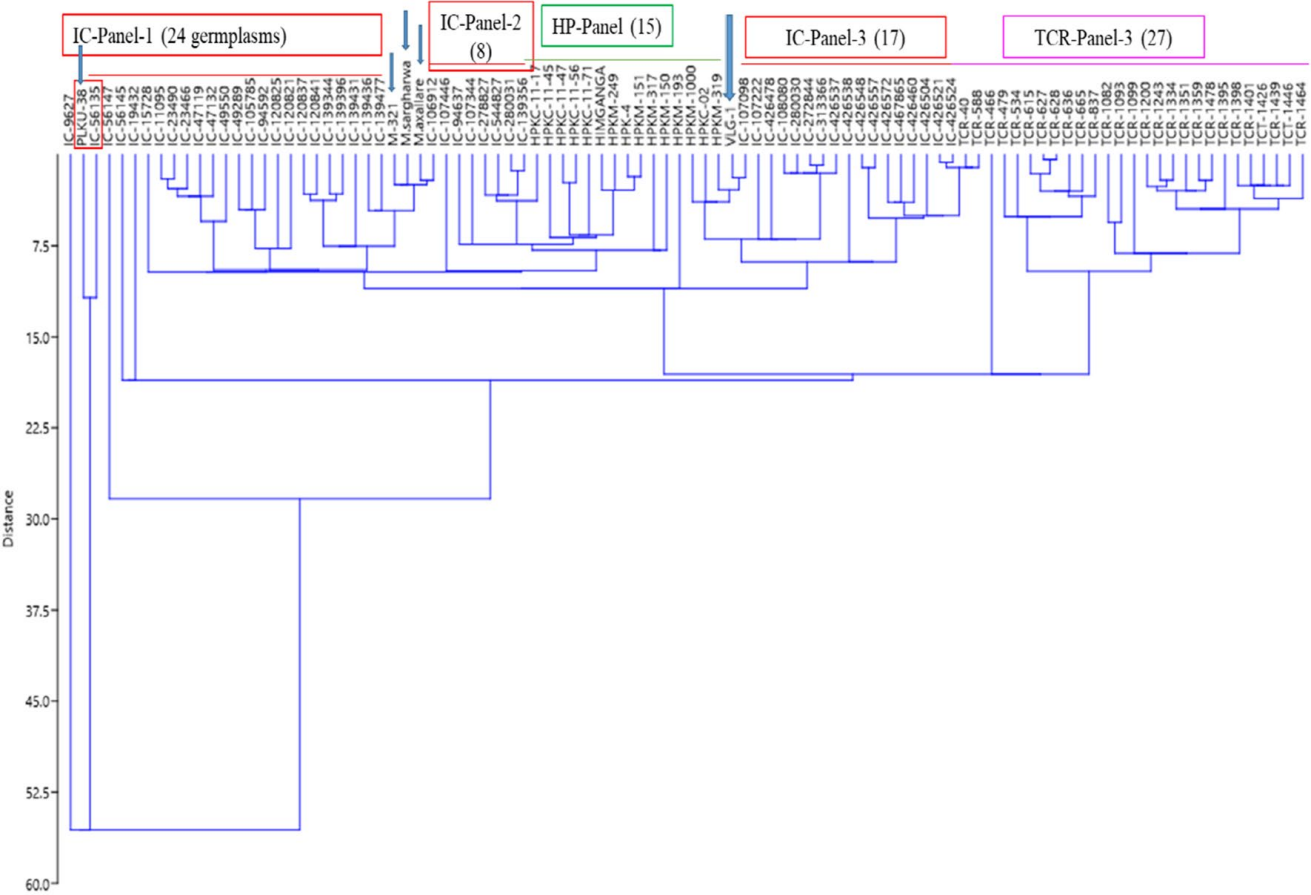
Hierarchical clustering among horsegram germplasm on the basis of ^1^H NMR data for the targeted metabolites.

^1^H NMR based information of selected panel of horsegram germplasm was statistically analyzed using PAST 4.0 software for the identification of lead chemotypes, similarities and differences among the diverse set of germplasm. Statistical analysis of large set of germplasm panel helps to visualize the clear discrimination through the correlations and variance studies. The Principal components and coordinate (PC and PCo), correspondence and hierarchical cluster (HC) analysis clearly showed the clusters (groups) and differentiations among the samples. Both, the PCA (Principal components analysis) and PCoA (Principal coordinates analysis) represented two major inliner cluster groups in the positive and negative plane with the variance of PC1, 30 germplasm were negatively correlated while 66 were positively correlated. Out of these one germplasm line (IC-19432) is positively correlated but situated in outliner that revealed its potential candidature for nutrition as it contains highest amount of targeted metabolites among the whole germplasms. Beside this, PKLU-38 and IC-56135 contained the least number of metabolites including only one flavonoid among the targeted metabolites (Figs. 3b and 4). These germplasm were strongly differentiated among the others. Over all % variance > 1% (Eigenvalue) was observed in the PC-1 to PC-5 as 56.61 (62.30), 20.93 (23.04), 9.59 (10.53), 6.24 (6.87), 1.17 (1.28) respectively. Further, qualitative and quantitative differences among the different lines were also observed from the various plots like PCA, PCoA and correspondence analysis of the diverse panel of horsegram germplasm (Fig. 4 and Figure SI-5), hierarchical clustering (Fig. 5), stacked bar plot (Figure SI-3) and matrix plot (Figure SI-4). The identified metabolites were classified on the basis of class of metabolites and their quantitative associations. The NMR metabolomics has shown locational variations as up and down regulation in metabolites were observed among the different cultivars. In view of the PCA and PCoA, the correspondence analysis on raw and column principal (Figure SI-5) also suggested that PLKU-38 and IC-56135 were different and does not fall in liner of the plot (Fig. 4). Furthermore, the similarities and differentiations and close associations of the germplasm were established with the hierarchical cluster analysis (HCA, Fig. 5). The IC Panel-I showed grouping of its 24 germplasm and also the association of its one germplasm (IC-56135) with PLKU-38. The lines M-321, *M. sar-gharwalensis and M. axillare* were associated with the IC-Panel’s while close association was observed with IC-106912. HP panel is group of 15 germplasm and associated with each other. They are also associated with the IC-panel-2 and VLG-1 germplasm. Further, VLG-1 is associated with the IC-Panel-3. The IC-Panel-3 is associated with TCR-Panel. The IC-Panel was found the highest germplasm containing group but observed with variations while the association group of the TCR was found largest among the 96 germplasm panel (Fig. 5). The immense metabolic diversity in plants is the direct result of continuous biological processes and adaptation to the changing climatic conditions. The adverse environment affects the plant growth, metabolism, physiological regulation and defense responses. On the other hand, abiotic stresses have an effect on the biogenesis, transport and storage of primary and secondary metabolites. A metabolic change in response to abiotic stress involves fine changes in organic compounds, formation of macromolecules, and other metabolic pathways. Correct activation of early metabolic responses helps the cells to restore chemical and energetic balances for the acclimatization.

## Conclusions

Metabolomics is the systematic approach through, which qualitative and chemical analysis of variety of metabolites present in the plants is done. Our data helped in the analysis of the diverse panel of 96 horsegram germplasm in terms of proteins and number of metabolites and helped to identify potential lines with high nutrient content. The variation among the whole germplasm was observed which may be due to the locational and varietal differences. Furthermore, this research can be beneficial to the plant breeders and provide new platform for the development of new varieties with high nutraceutical value. The various metabolites (like terpenoids, phenolics, alkaloids, flavonoids, saponins, carotenoids, tannins and soluble/insoluble dietary fibers) are the main health promoting bioagents with potent antioxidant properties^26^. Variation in metabolite levels among the seeds of 96 lines of horsegram germplasm (up and down regulation) were observed, which highlights the plant-biotic/abiotic relationships during a specific environment. The findings could also be exploited for further agriculture interventions especially in breeding to get substantial amounts of bioactive ingredients, to understand the impact of environmental factors on the assembly of metabolites, to develop the chemical signature, for the characterization of plant ecotypes and methods to know the biological diversity of horsegram and its products.

## Supplementary Information

Supplementary Information.
