# Supplementary material for: Metabolite profiling and protein quantification to a large library of 96 horsegram (Macrotyloma uniflorum) germplasm
Source: Sci Rep. 2022 May 12;12:7865. doi: 10.1038/s41598-022-11962-7 (PMC9098527; doi:10.1038/s41598-022-11962-7)
Supplement: Supplementary file 1 — Supplementary Information. [file 41598_2022_11962_MOESM1_ESM.doc]

**SUPPLEMENTARY MATERIAL**

Metabolite profiling and protein quantification to a large library of 96 horsegram (*Macrotyloma uniflorum*) germplasm

Manisha Gautam^1^ and Rakesh Kumar Chahota^1*^

^1^Department of Agricultural Biotechnology, CSK Himachal Pradesh Agricultural University, Palampur, India

**Figures**

**SI-1**Nuclear magnetic resonance spectral for diverse panel of horsegram germplasm [(a)from 11-50 lines, (b)from 51-90 lines, (c)from 91-96 lines]

**SI-2**Two dimensional NMR techniques:(a) HSQC (Heteronuclear Single Quantum Coherence), (b) COSY (Correlated Spectroscopy),(c) HMBC (Heteronuclear Multiple Bond Coherence), and (d) NOESY (Nuclear Overhauser Effect Spectroscopy)

**SI-3** Stacked bar plot for the targeted metabolites of diverse panel of horsegram germplasm

**SI-4** Matrix plot for the targeted metabolites of diverse panel of horsegram germplasm

**SI-5** Correspondence analysis of the diverse panel of horsegram germplasm of targeted metabolites

**Tables**

**SI-1:** Diverse panel of horsegram germplasm

**SI-2:** ^1^H NMR chemical shift data of diverse panel of horsegram germplasm for all identified metabolites (mg/g)


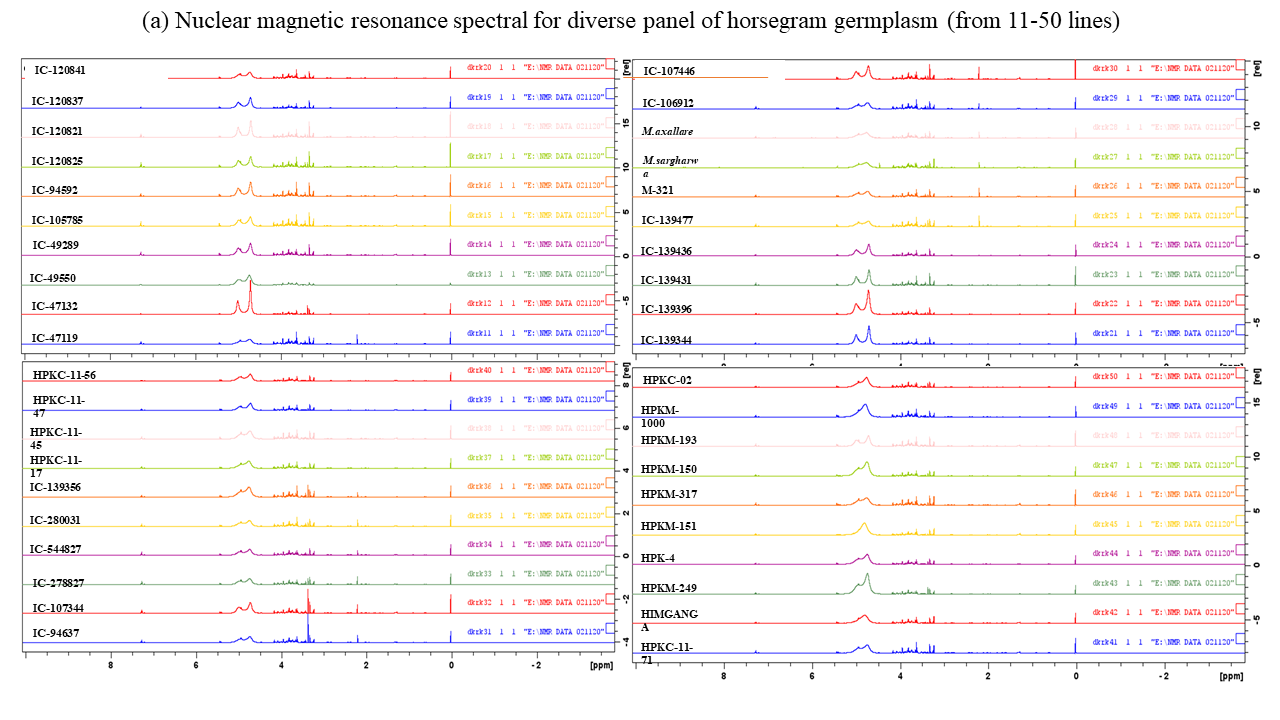


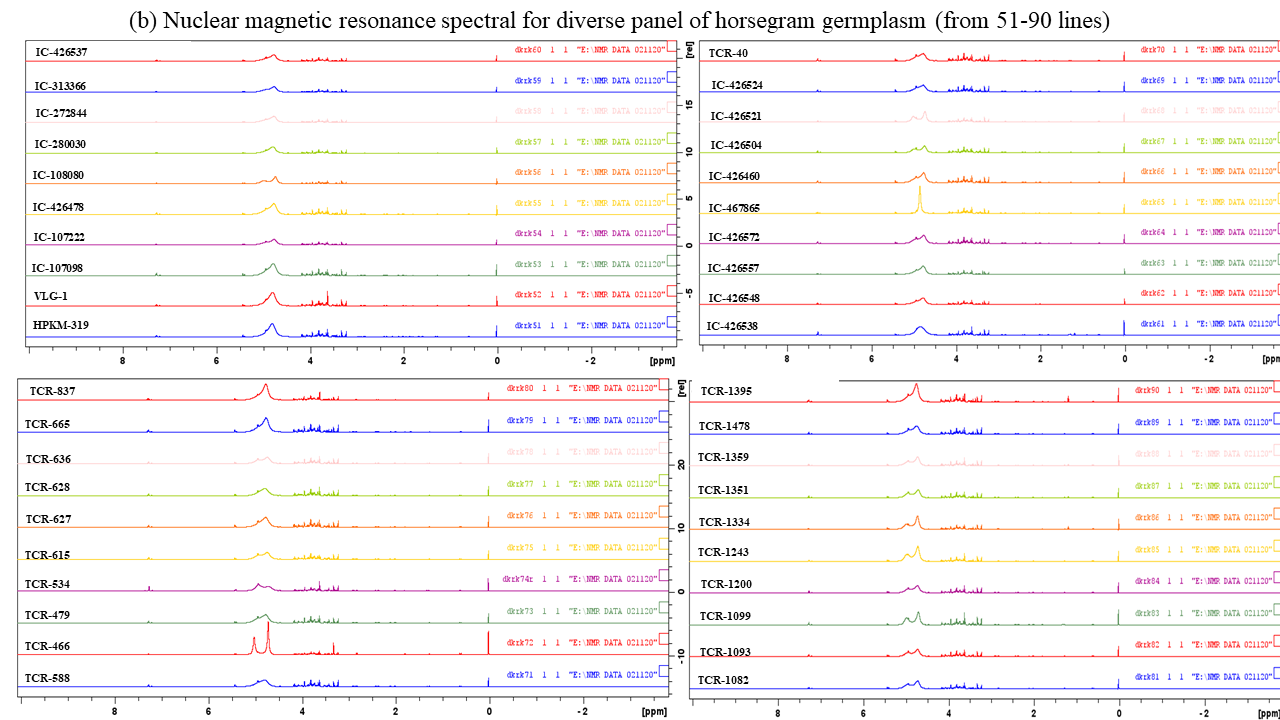


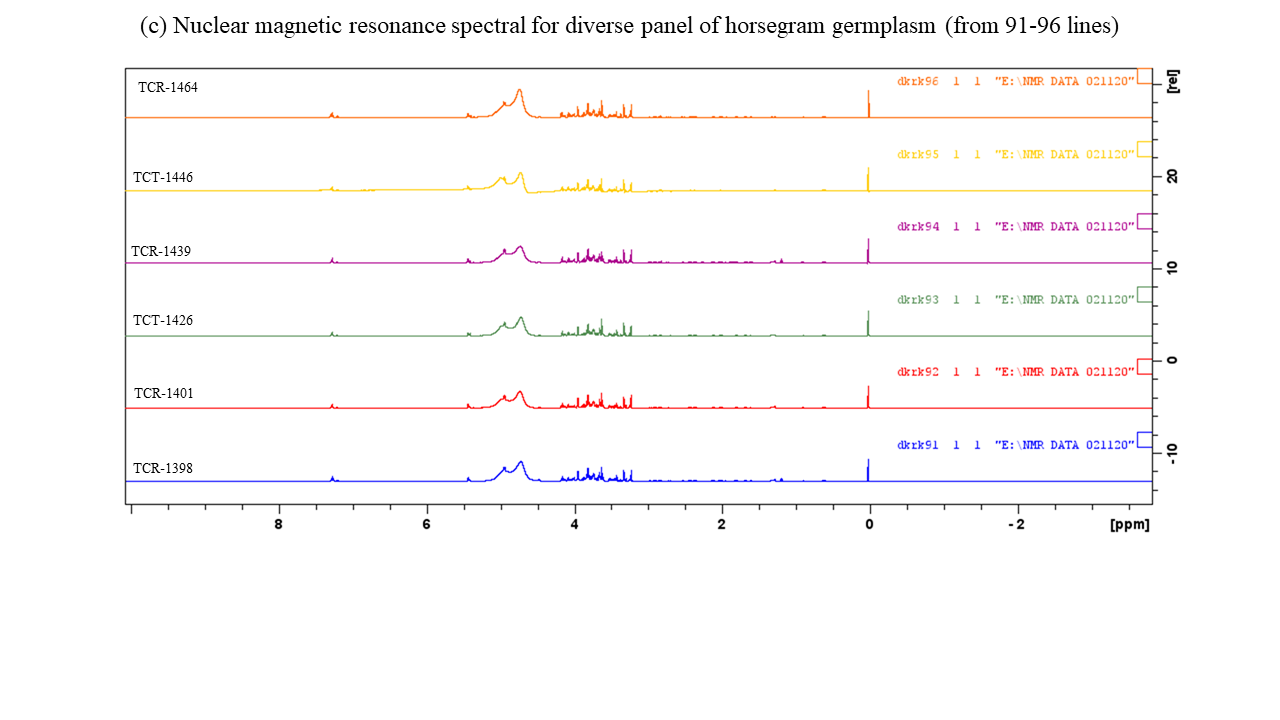


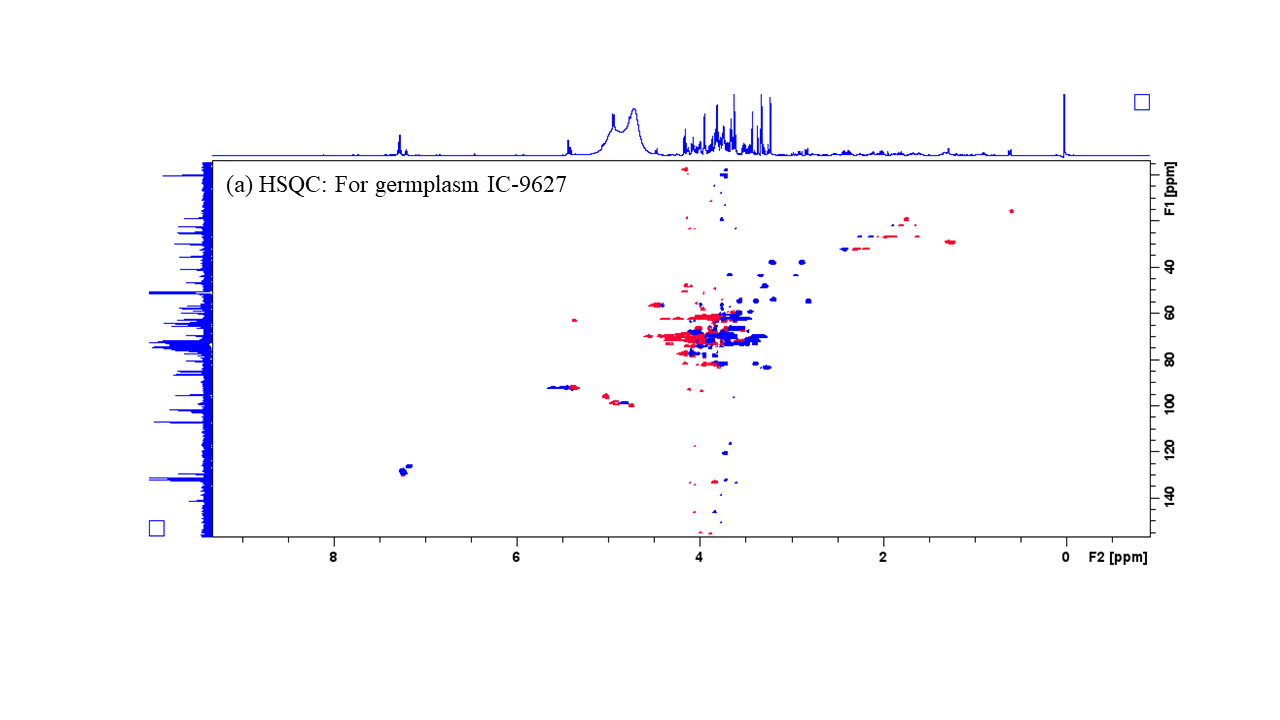


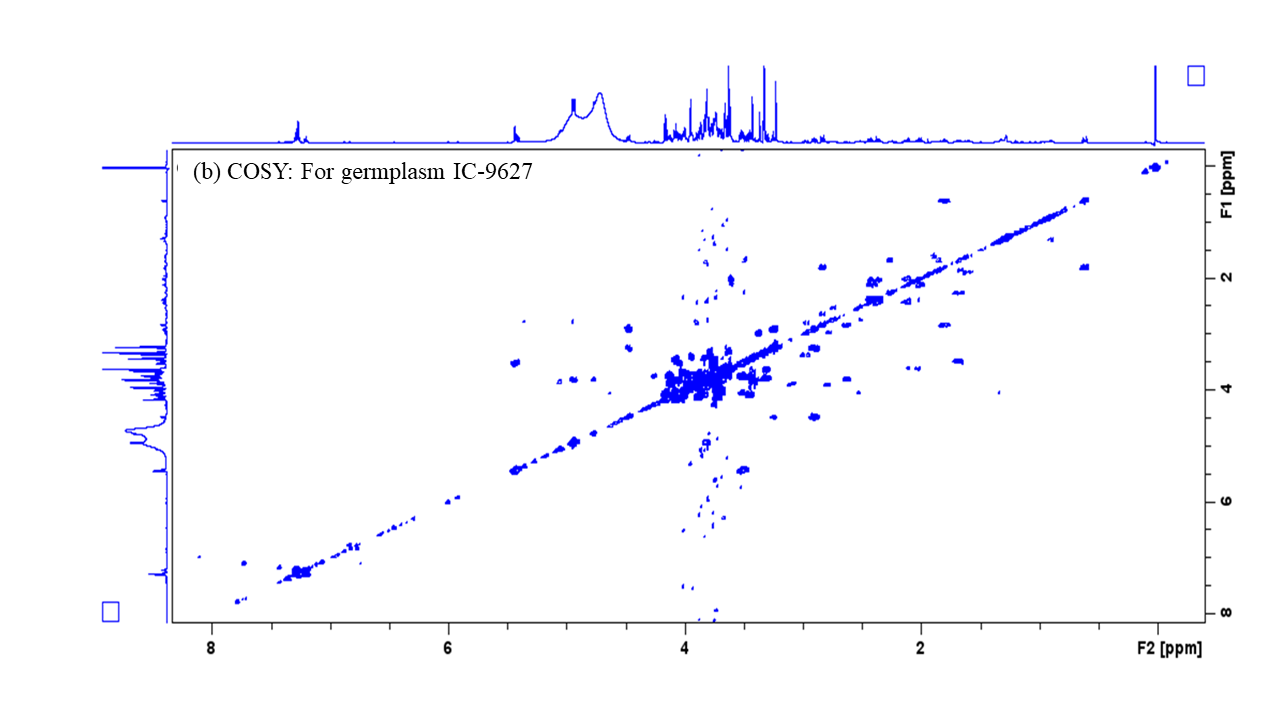


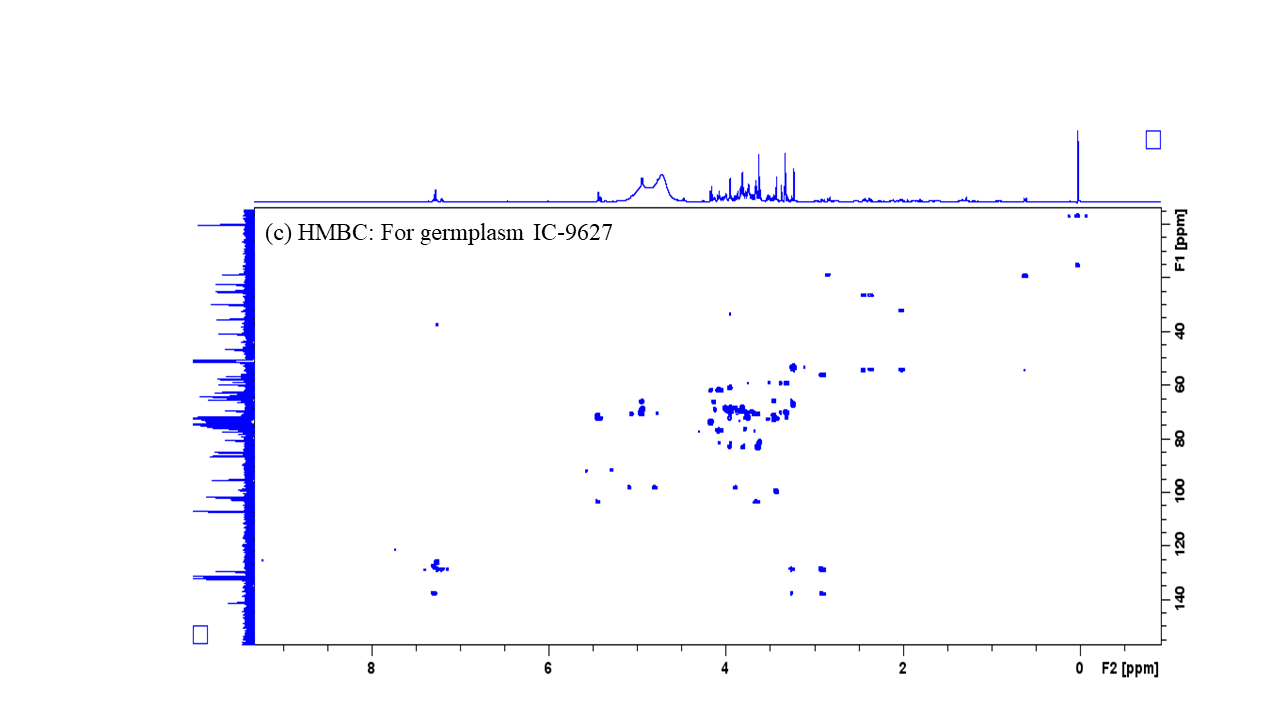


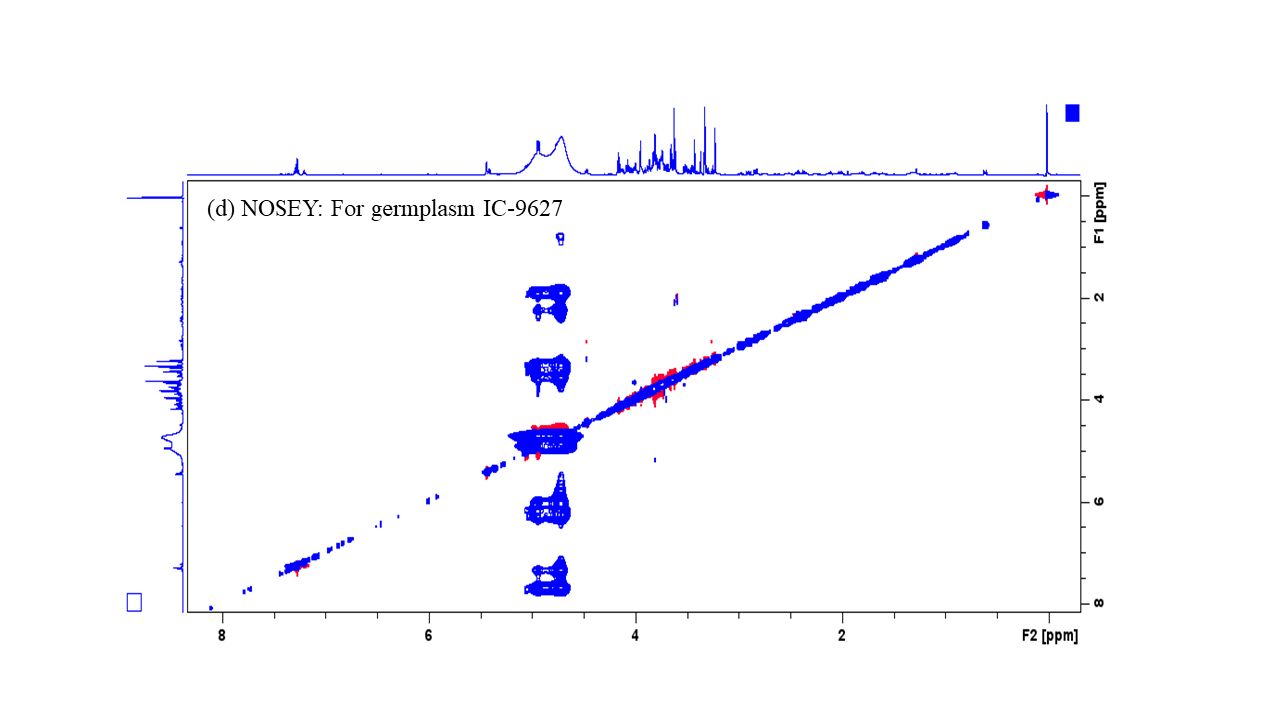


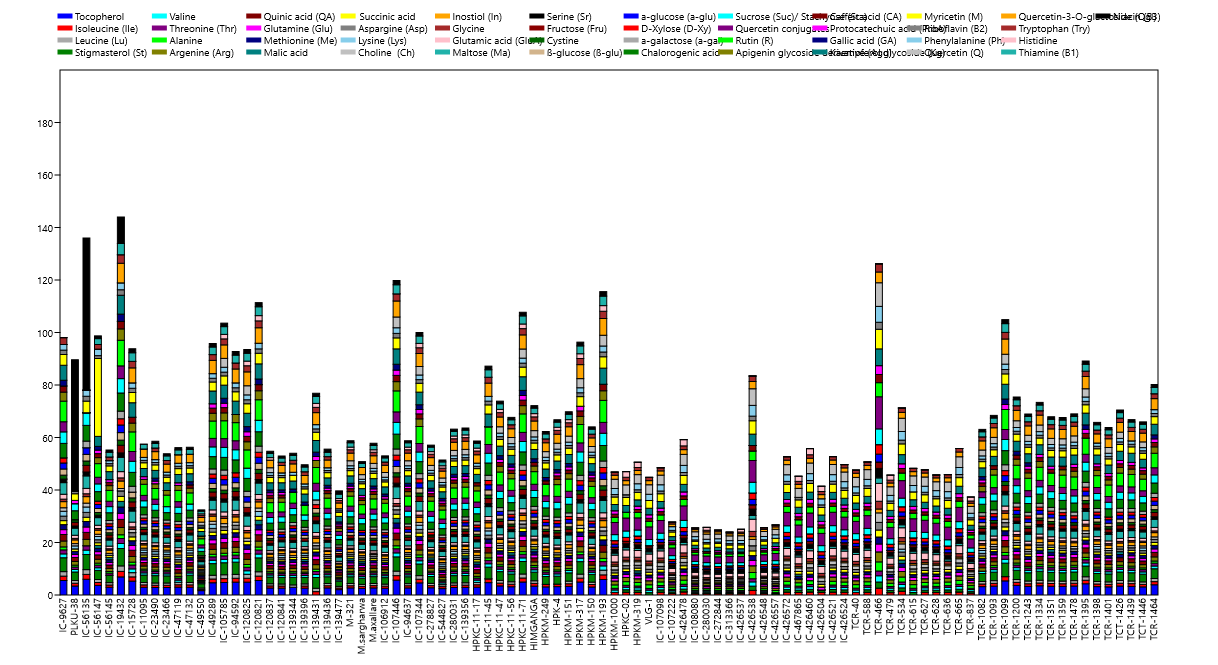


**SI-3** Stacked bar plot for the targeted metabolites of diverse panel of horsegram germplasm


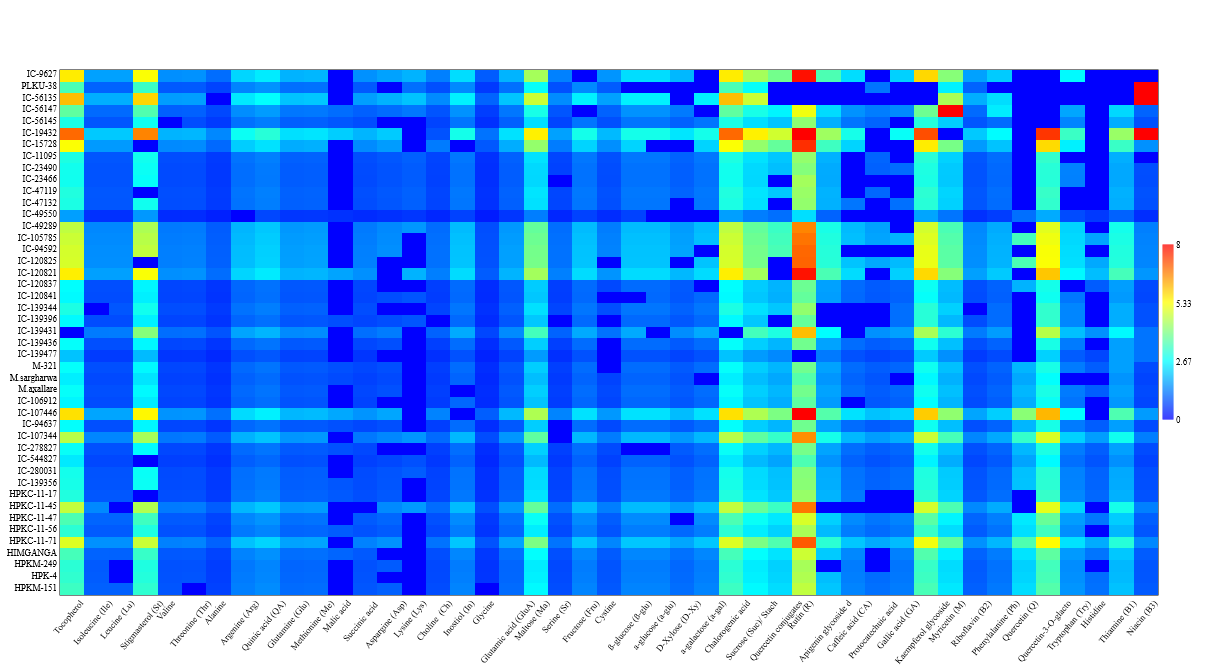


**SI-4**Matrix plot for the targeted metabolites of diverse panel of horsegram germplasm


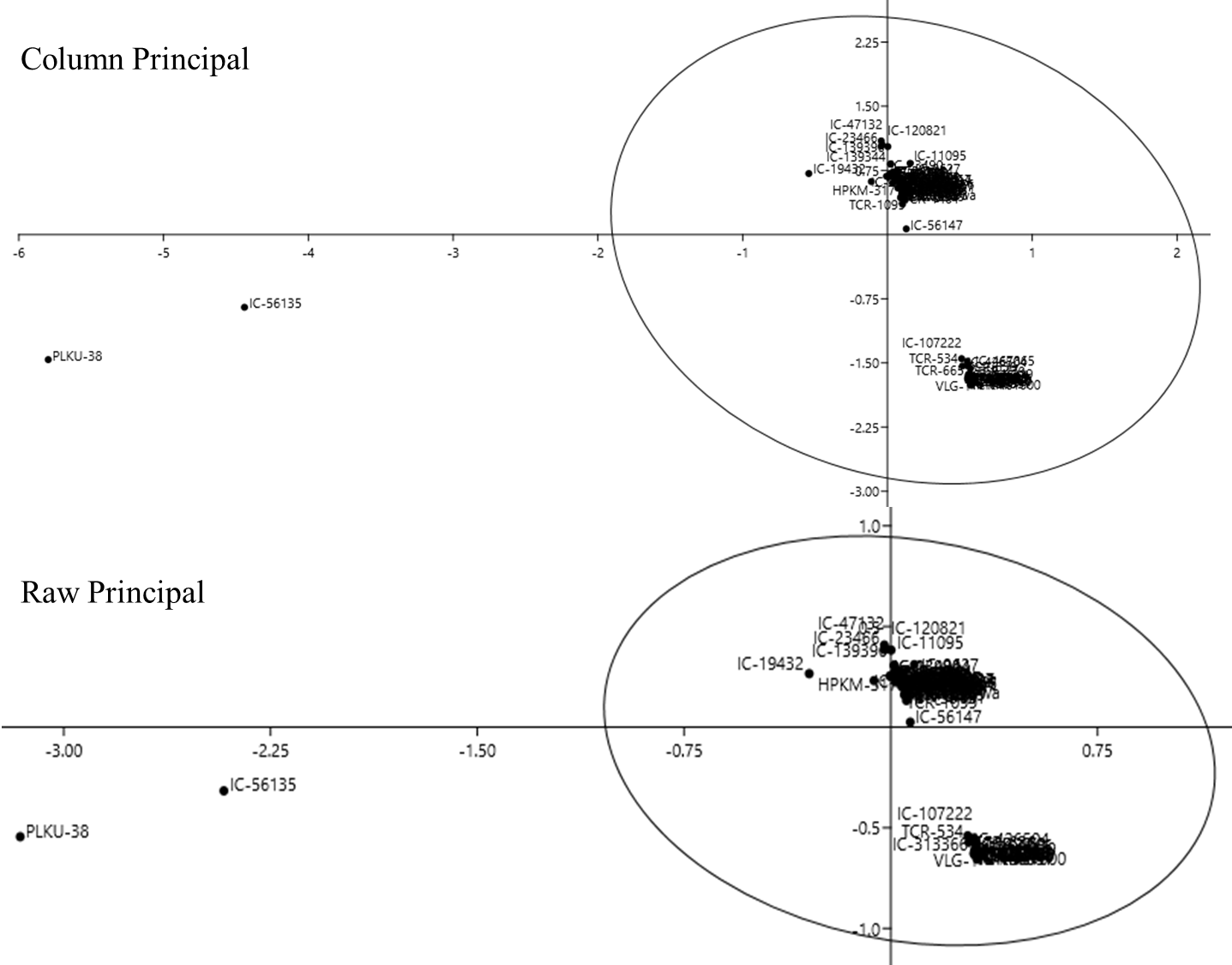


**SI-5** Correspondence analysis of the diverse panel of horsegram germplasm of targeted metabolites

**SI-1:** Diverse panel of horsegram germplasm

| **Sr. No.** | **IC No.** | **Location** |
| --- | --- | --- |
| 1 | IC-9627 | Sikkim, India |
| 2 | PLKU-38 | Himachal Pradesh, India |
| 3 | IC-56135 | Andhra Pradesh, India |
| 4 | IC-56147 | Andhra Pradesh, India |
| 5 | IC-56145 | Andhra Pradesh, India |
| 6 | IC-19432 | Manipur/Odisha, India |
| 7 | IC-15728 | Bihar/Jharkhand, India |
| 8 | IC-11095 | Punjab, India |
| 9 | IC-23490 | Madhya Pradesh, India |
| 10 | IC-23466 | Madhya Pradesh, India |
| 11 | IC-47119 | Madhya Pradesh, India |
| 12 | IC-47132 | Andhra Pradesh, India |
| 13 | IC-49550 | Maharashtra, India |
| 14 | IC-49289 | Maharashtra, India |
| 15 | IC-105785 | Bihar, India |
| 16 | IC-94592 | Bihar, India |
| 17 | IC-120825 | Madhya Pradesh, India |
| 18 | IC-120821 | Madhya Pradesh, India |
| 19 | IC-120837 | Punjab, India |
| 20 | IC-120841 | Madhya Pradesh, India |
| 21 | IC-139344 | Maharashtra, India |
| 22 | IC-139396 | Rajasthan, India |
| 23 | IC-139431 | Delhi, India |
| 24 | IC-139436 | Rajasthan, India |
| 25 | IC-139477 | Rajasthan, India |
| 26 | M-321 | Himachal Pradesh, India |
| 27 | *M.sar-gharwalensis* | Uttarakhand, India |
| 28 | *M.axillare* | Australia |
| 29 | IC-106912 | Doda |
| 30 | IC-107446 | Pithoragarh, Uttarakhand |
| 31 | IC-94637 | Uttarkashi, Uttarakhand |
| 32 | IC-107344 | Punjab |
| 33 | IC-278827 | Sirmour, Himachal pradesh |
| 34 | IC-544827 | Shimla, Himachal Pradesh |
| 35 | IC-280031 | Chamba, Himachal Pradesh |
| 36 | IC-139356 | Rajasthan, India |
| 37 | HPKC-11-17 | Himachal Pradesh, India |
| 38 | HPKC-11-45 | Himachal Pradesh, India |
| 39 | HPKC-11-47 | Himachal Pradesh, India |
| 40 | HPKC-11-56 | Himachal Pradesh, India |
| 41 | HPKC-11-71 | Himachal Pradesh, India |
| 42 | HIMGANGA | Himachal Pradesh, India |
| 43 | HPKM-249 | Himachal Pradesh, India |
| 44 | HPK-4 | Himachal Pradesh, India |
| 45 | HPKM-151 | Himachal Pradesh, India |
| 46 | HPKM-317 | Himachal Pradesh, India |
| 47 | HPKM-150 | Himachal Pradesh, India |
| 48 | HPKM-193 | Himachal Pradesh, India |
| 49 | HPKM-1000 | Himachal Pradesh, India |
| 50 | HPKC-02 | Himachal Pradesh, India |
| 51 | HPKM-319 | Himachal Pradesh, India |
| 52 | VLG-1 | Uttarakhand, India |
| 53 | IC-107098 | Andhra Pradesh, India |
| 54 | IC-107222 | Andhra Pradesh, India |
| 55 | IC-426478 | Andhra Pradesh, India |
| 56 | IC-108080 | Andhra Pradesh, India |
| 57 | IC-280030 | Madhya Pradesh |
| 58 | IC-272844 | Karnataka, India |
| 59 | IC-313366 | Karnataka, India |
| 60 | IC-426537 | Karnataka, India |
| 61 | IC-426538 | Madhya Pradesh |
| 62 | IC-426548 | Andhra Pradesh, India |
| 63 | IC-426557 | Telangana, India |
| 64 | IC-426572 | Andhra Pradesh, India |
| 65 | IC-467865 | Punjab, India |
| 66 | IC-426460 | Karnataka, India |
| 67 | IC-426504 | Andhra Pradesh, India |
| 68 | IC-426521 | Andhra Pradesh, India |
| 69 | IC-426524 | Sikkim, India |
| 70 | TCR-40 | Andhra Pradesh, India |
| 71 | IC-71812 (TCR-588) | Tamil Nadu, India |
| 72 | IC-68593 (TCR-466) | Kerala, India |
| 73 | IC-68606 (TCR-479) | Kerala, India |
| 74 | TCR-534 | Andhra Pradesh, India |
| 75 | TCR-615 | Andhra Pradesh, India |
| 76 | TCR-627 | Andhra Pradesh, India |
| 77 | TCR-628 | Andhra Pradesh, India |
| 78 | TCR-636 | Andhra Pradesh, India |
| 79 | TCR-665 | Andhra Pradesh, India |
| 80 | TCR-837 | Andhra Pradesh, India |
| 81 | TCR-1082 | Andhra Pradesh, India |
| 82 | TCR-1093 | Andhra Pradesh, India |
| 83 | TCR-1099 | Andhra Pradesh, India |
| 84 | TCR-1200 | Andhra Pradesh, India |
| 85 | TCR-1243 | Andhra Pradesh, India |
| 86 | TCR-1334 | Andhra Pradesh, India |
| 87 | TCR-1351 | Andhra Pradesh, India |
| 88 | TCR-1359 | Andhra Pradesh, India |
| 89 | TCR-1478 | Andhra Pradesh, India |
| 90 | TCR-1395 | Andhra Pradesh, India |
| 91 | TCR-1398 | Andhra Pradesh, India |
| 92 | TCR-1401 | Andhra Pradesh, India |
| 93 | TCT-1426 | Andhra Pradesh, India |
| 94 | TCR-1439 | Andhra Pradesh, India |
| 95 | TCT-1446 | Andhra Pradesh, India |
| 96 | TCR-1464 | Andhra Pradesh, India |


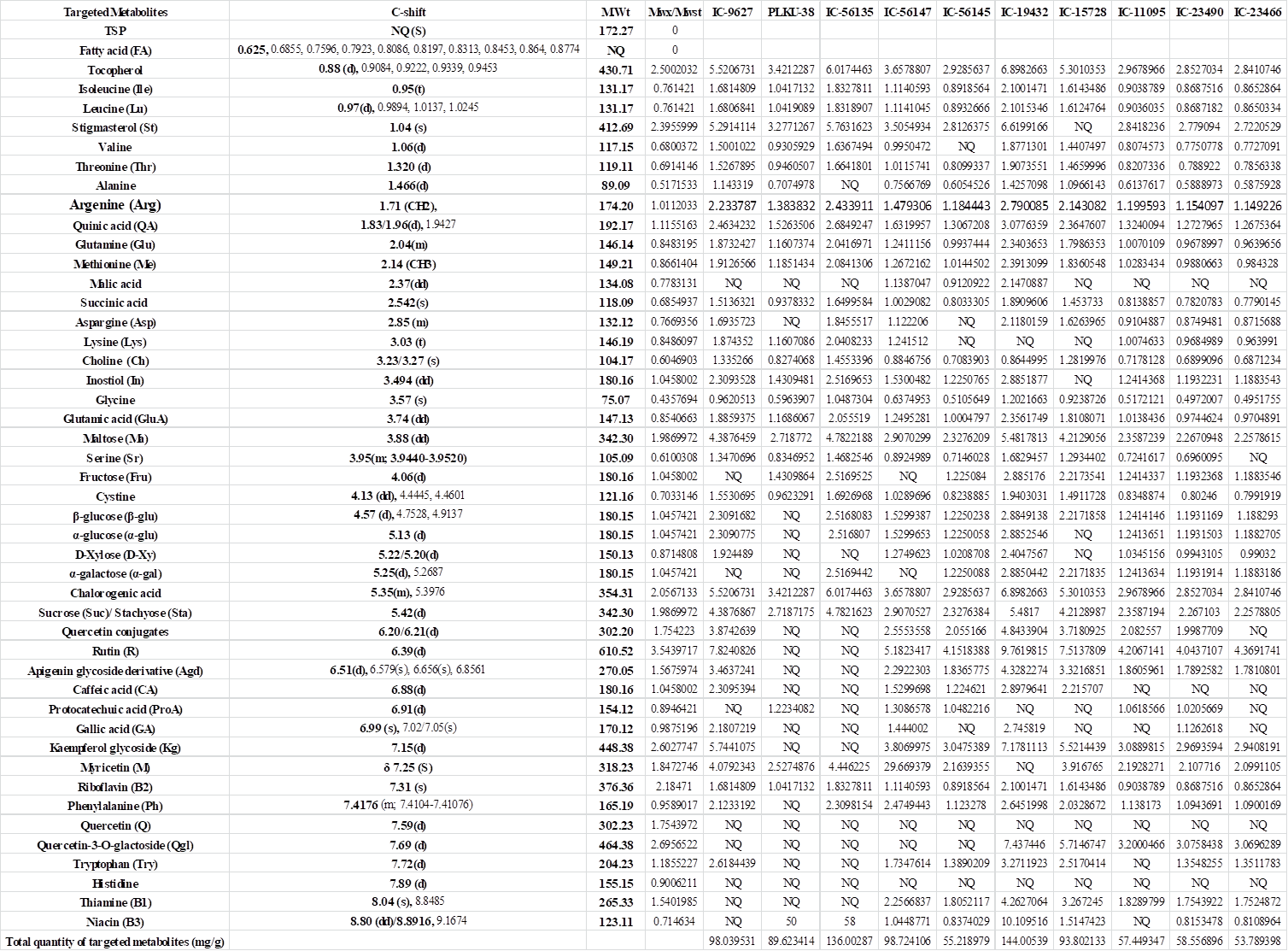
*NQ= Not Quantifiable

**Table SI-2:** ^1^H NMR chemical shift data of diverse panel of horsegram germplasm for all identified metabolites (mg/g)


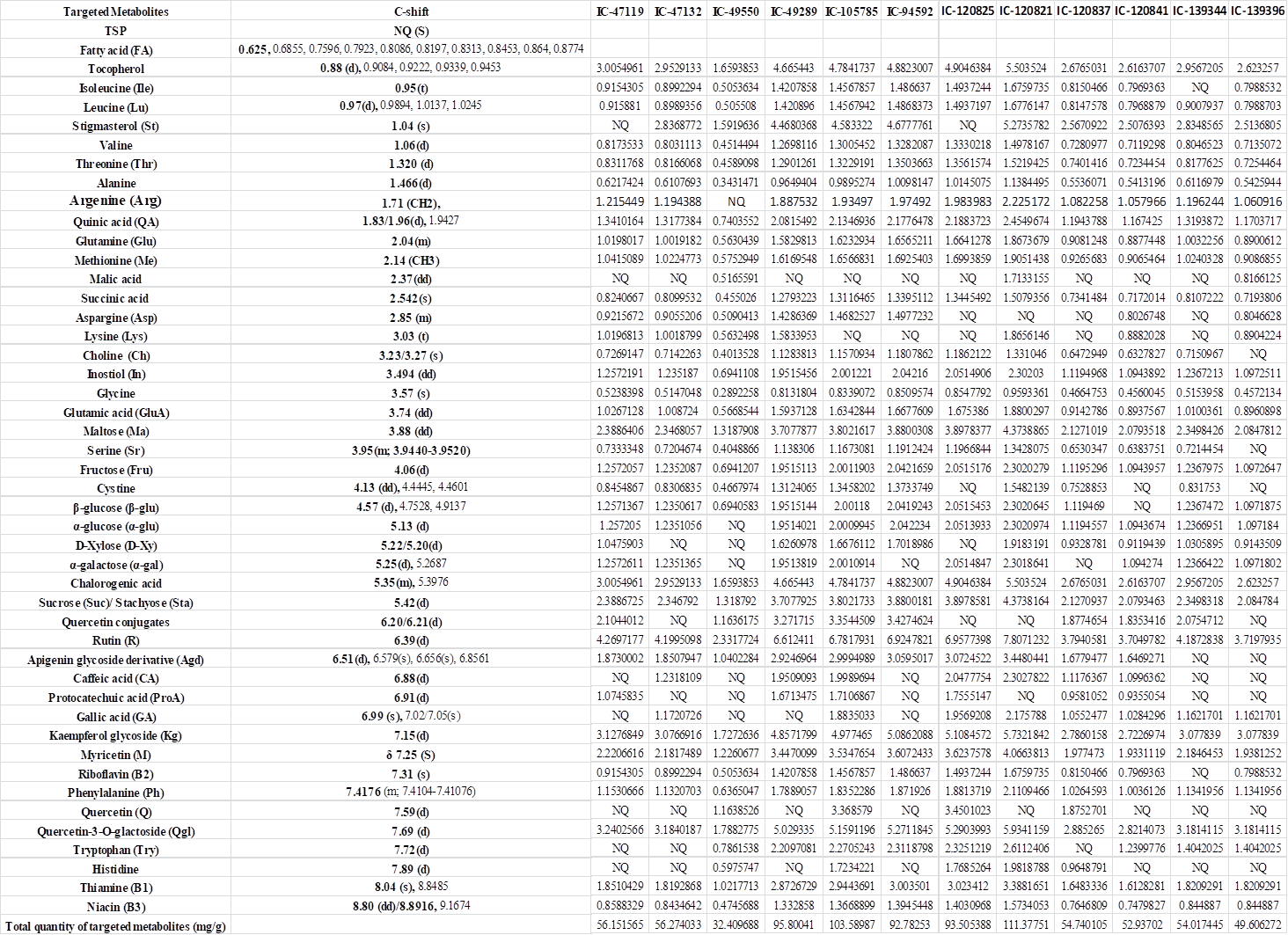
*NQ= Not Quantifiable


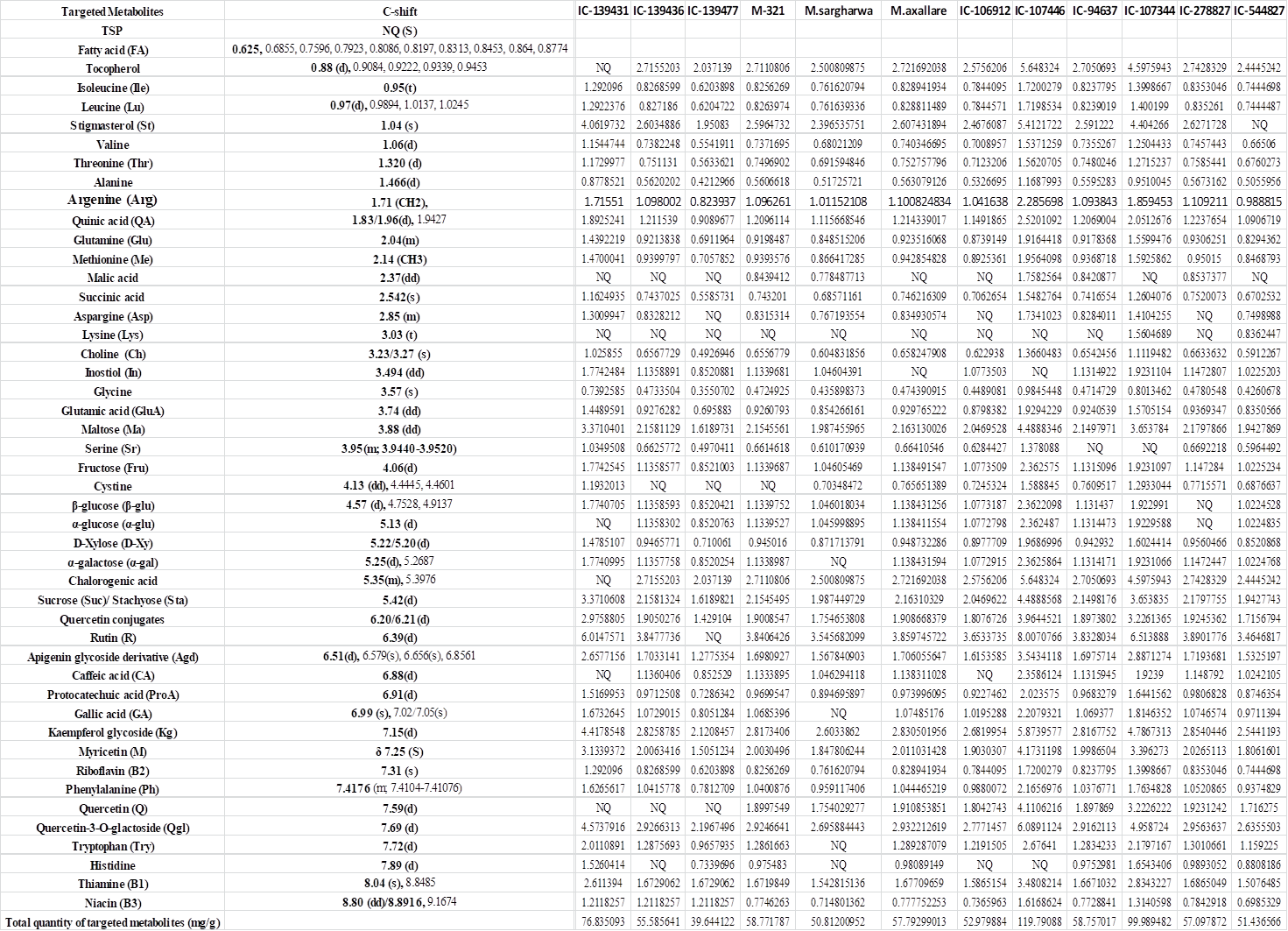
*NQ= Not Quantifiable


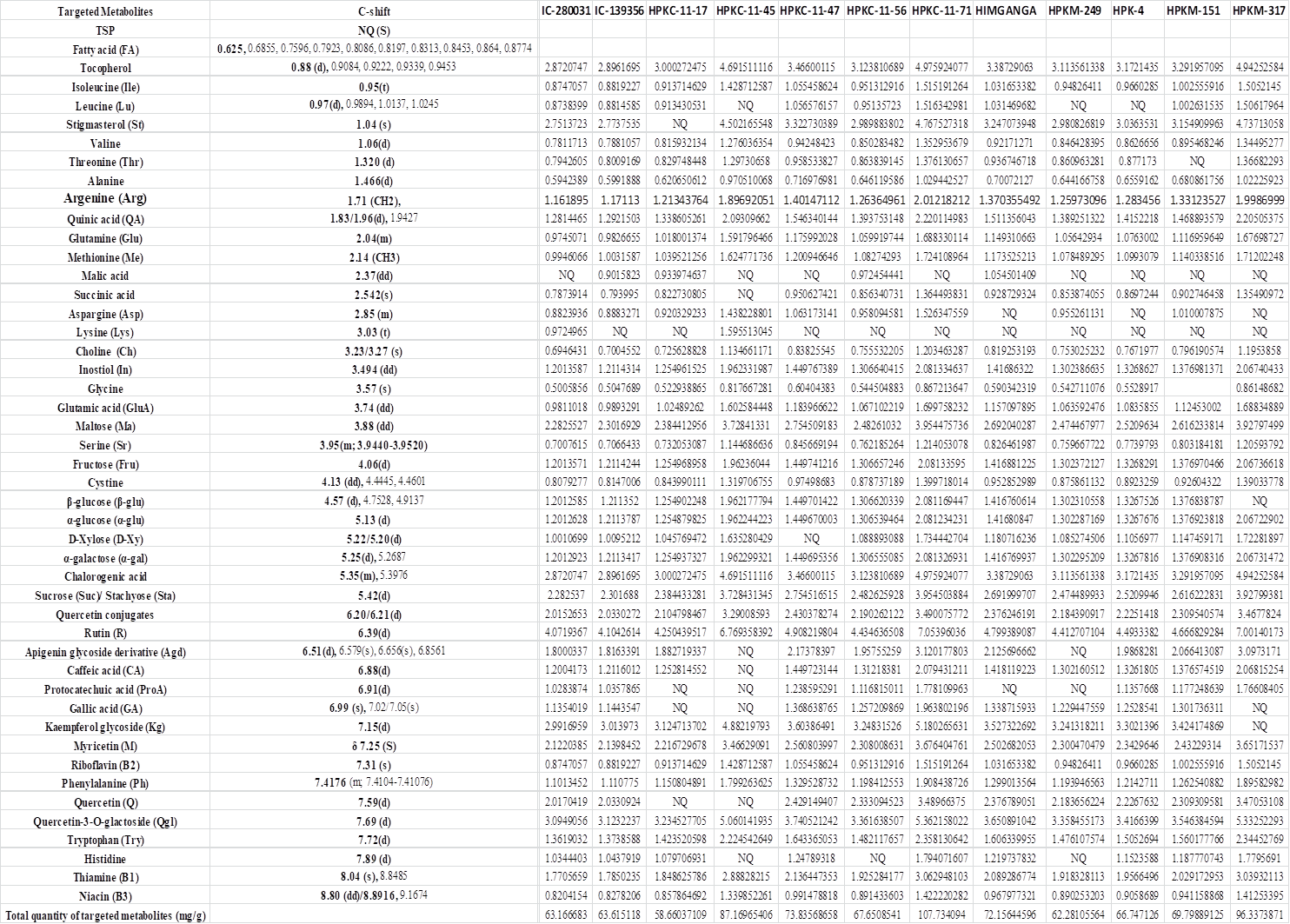
*NQ= Not Quantifiable


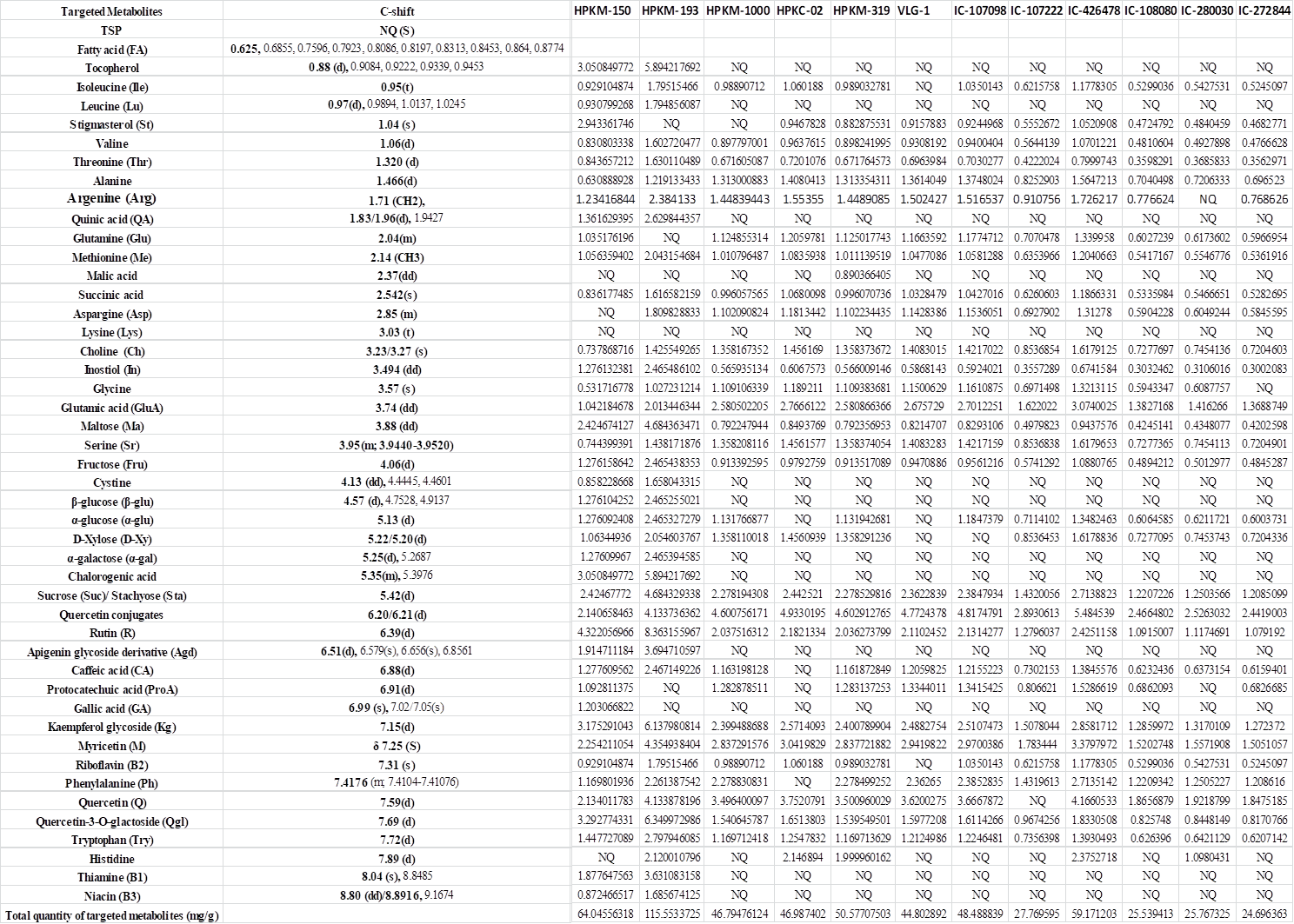
*NQ= Not Quantifiable


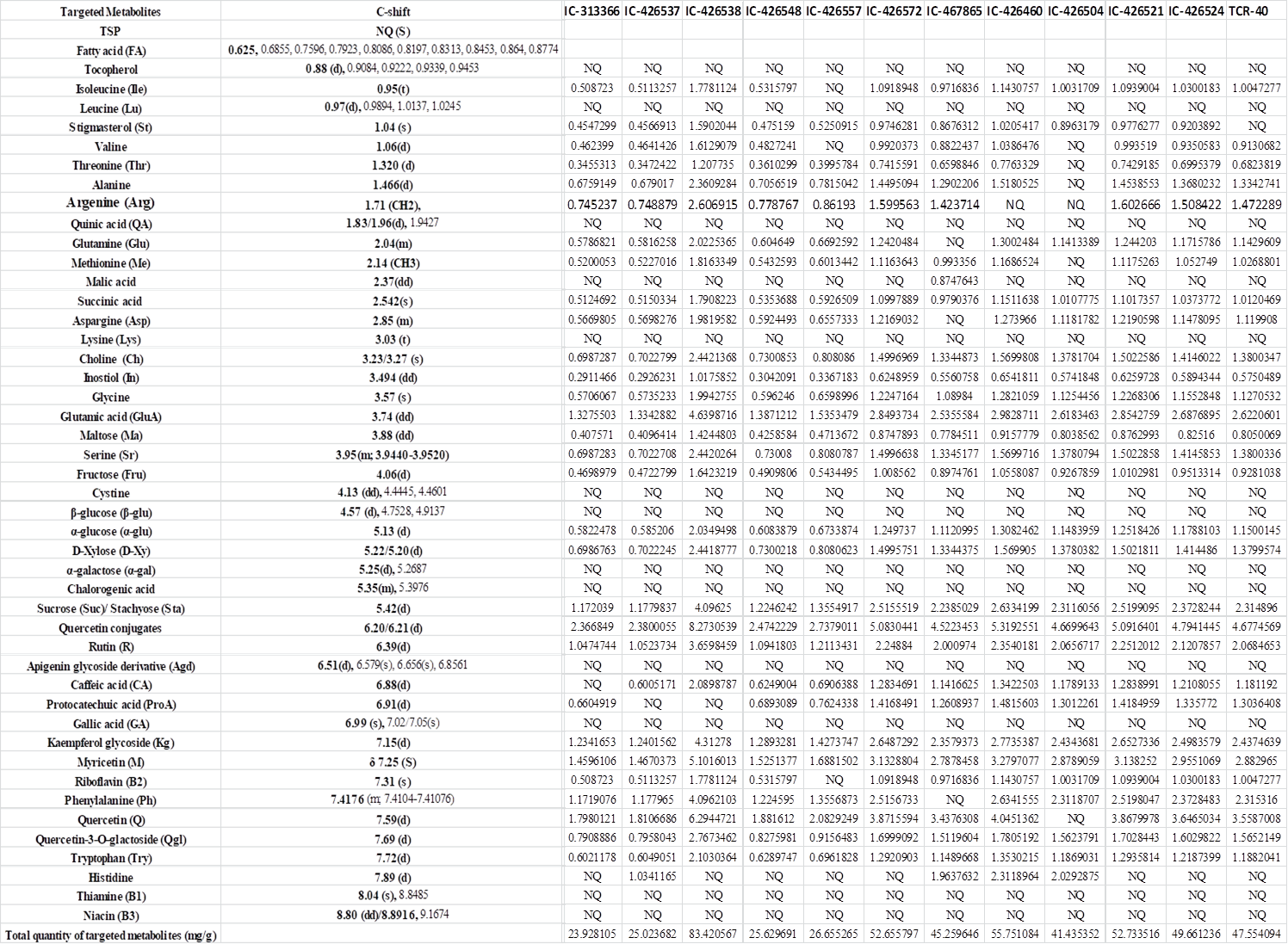
*NQ= Not Quantifiable


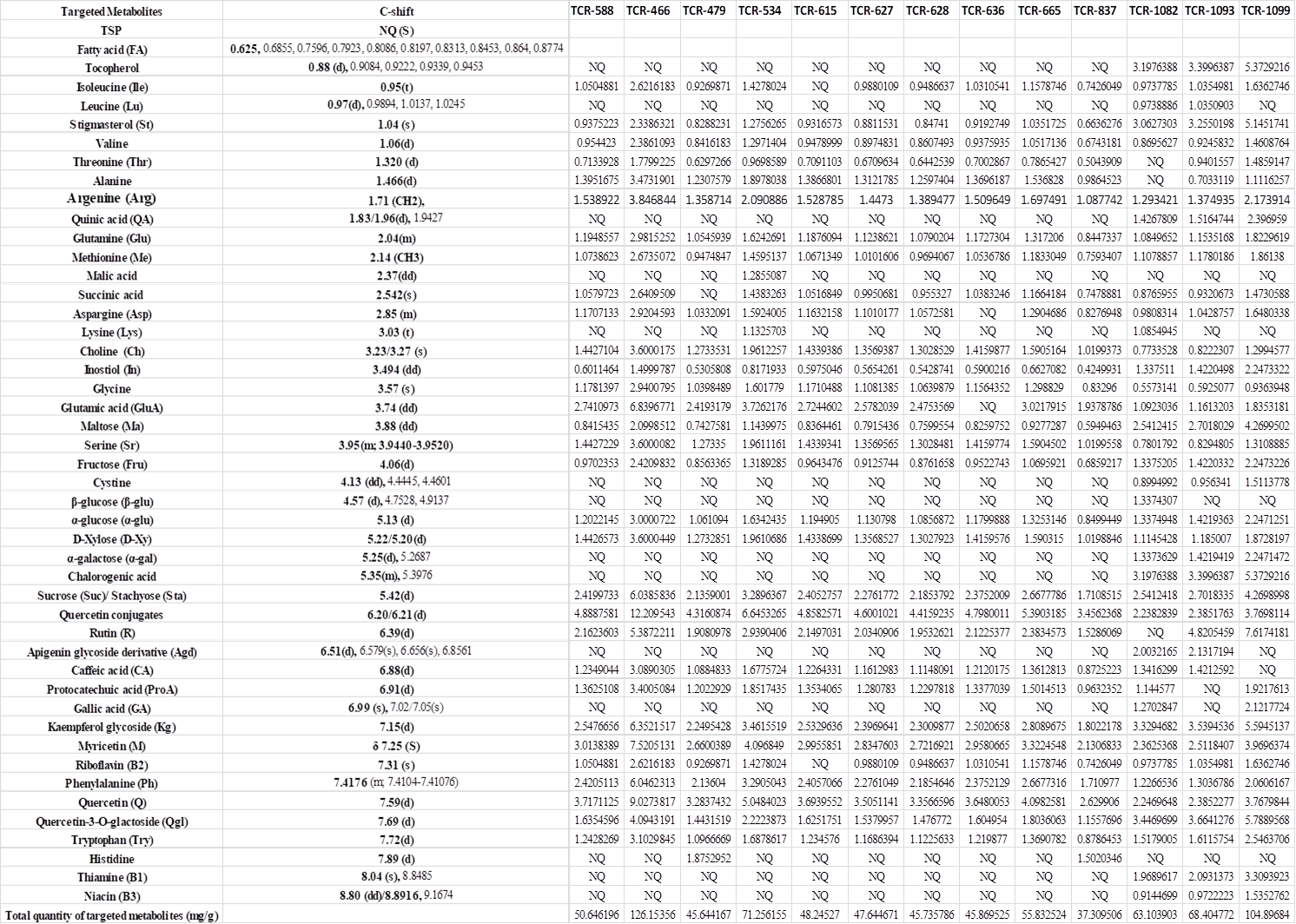
*NQ= Not Quantifiable


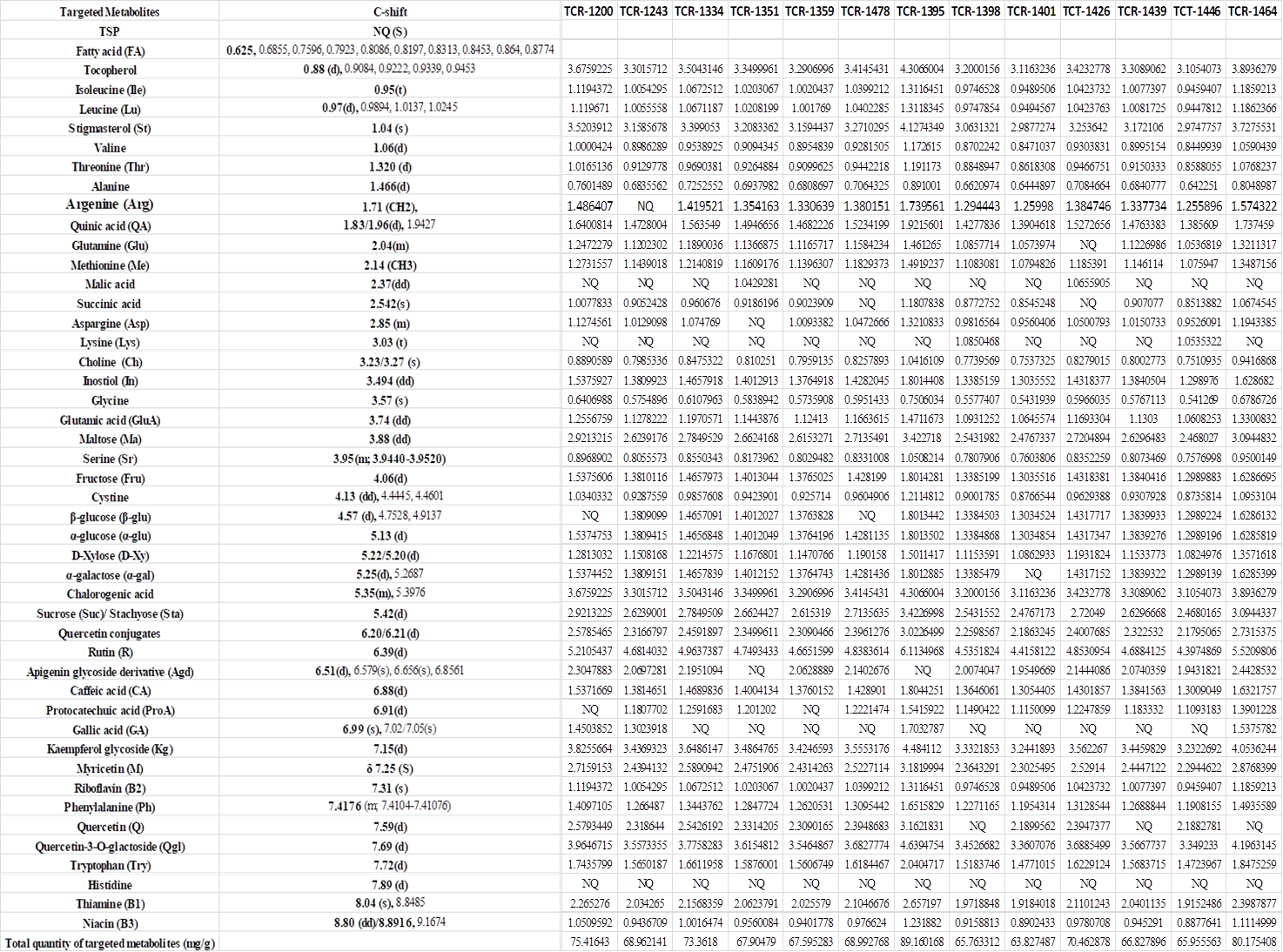
*NQ= Not Quantifiable
